# High Diversity Revealed in Leaf‐Associated Protists (Rhizaria: Cercozoa) of Brassicaceae

**DOI:** 10.1111/jeu.12314

**Published:** 2016-04-14

**Authors:** Sebastian Ploch, Laura E. Rose, David Bass, Michael Bonkowski

**Affiliations:** ^1^ Institute of Population Genetics, Cluster of Excellence in Plant SciencesUniversity of DüsseldorfUniversitätsstr. 1D‐40225DüsseldorfGermany; ^2^ Senckenberg Biodiversity and Climate Research CenterGeorg‐Voigt‐Street 14‐16D‐60325FrankfurtGermany; ^3^ Department of Life SciencesThe Natural History MuseumLondonSW7 5BDUnited Kingdom; ^4^ Centre for Environment, Fisheries, and Aquaculture Science (Cefas)Barrack RoadThe NotheWeymouthDorsetDT4 8UBUnited Kingdom; ^5^ Department of Terrestrial EcologyInstitute of ZoologyUniversity of CologneZülpicher Street 47bD‐50674KölnGermany

**Keywords:** 18S, *Arabidopsis*, Cercomonas, cloning, environmental sequencing, phyllosphere, protist

## Abstract

The largest biological surface on earth is formed by plant leaves. These leaf surfaces are colonized by a specialized suite of leaf‐inhabiting microorganisms, recently termed “phyllosphere microbiome”. Microbial prey, however, attract microbial predators. Protists in particular have been shown to structure bacterial communities on plant surfaces, but virtually nothing is known about the community composition of protists on leaves. Using newly designed specific primers targeting the 18S rDNA gene of Cercozoa, we investigated the species richness of this common protist group on leaves of four Brassicaceae species from two different locations in a cloning‐based approach. The generated sequences revealed a broad diversity of leaf‐associated Cercozoa, mostly bacterial feeders, but also including known plant pathogens and a taxon of potential endophytes that were recently described as algal predators in freshwater systems. This initial study shows that protists must be regarded as an integral part of the microbial diversity in the phyllosphere of plants.

PLANT leaves are estimated to form the largest biological surface on Earth with an area exceeding 10^8^ km² globally (Penuelas and Terradas 2014). Bacteria were found to be the dominant leaf colonizers with numbers often exceeding 10^7^ cells/cm^2^ leaf surface (Lindow and Leveau 2002). Bacteria offer a rich food source for predators on leaf surfaces, and we have good reasons to assume that microbial food webs are the rule rather than the exception in the plant phyllosphere. In particular, protists are well‐known predators on plant surfaces (Bonkowski 2004; Rosenberg et al. 2009), but systematic taxonomic studies on the diversity of phyllosphere protists are scarce (Bamforth 1973).

There is evidence that some protists are adapted for life in the phyllosphere (Bamforth 1973). For example, Mueller and Mueller (1970) described in detail the diurnal life of *Colpoda cucullus*, a ciliate commonly preying and multiplying on plant leaves. Laboratory experiments showed that *C. cucullus* reduced the numbers of *Pseudomonas syringae* on bean leaves by two orders of magnitude (Lindow 2006), giving evidence that bacterial phyllosphere communities might not only be structured by interspecific competition, plant characteristics, or the harsh abiotic environment (De Costa et al. 2006; Kinkel 1997), but also by a substantial predation pressure from protists.

The presence of a broad variety of protist taxa on plant leaves is well documented, but phyllosphere protists have been mainly studied with respect to food safety, either as potential vectors of pathogenic bacteria or as potential human pathogens (Ciurea‐Van Saanen 1981; Gourabathini et al. 2008; Napolitano 1982; Napolitano and Colletti‐Eggolt 1984; Rude et al. 1983; Vaerewijck and Houf 2014; Vaerewijck et al. 2011). No molecular study targeting phyllosphere protists has been conducted to date.

Protists are exceptionally diverse (Burki 2014), and therefore “general” eukaryotic primers fail to target a great majority of protist taxa. Since some “general” eukaryotic primers have the additional disadvantage to preferentially amplify fungal taxa, and are biased towards particular protist lineages (Adl et al. 2014; Lentendu et al. 2014), we chose a targeted approach with a focus on Cercozoa. Cercozoa are a monophyletic, highly diverse, and dominant group of protists in terrestrial systems (Bass et al. 2005; Geisen et al. 2015; Urich et al. 2008). Cercozoan bacterivorous flagellates and amoeboflagellates are abundant and diverse in many environments (Bass et al. 2009a,b; Howe et al. 2009). Some cercozoans have been shown to withstand environmental extremes (Hughes and Smith 1989); and their ability to rapidly excyst, feed, and multiply within hours (Ekelund 1996) appears to be a particularly well‐suited adaptation to the fluctuating environmental conditions in the phyllosphere. Furthermore, Cercozoa contain potential endophytic lineages (e.g. Neuhauser et al. 2014). Some cercozoan taxa evolved mechanisms to penetrate plant cell walls (Hess and Melkonian 2013), whereas other lineages contain well‐known plant pathogens such as *Plasmodiophora brassicae*, the agent of club‐root disease in Brassicaceae (Neuhauser et al. 2014).

Brassicaceae include important crop plants including oilseed rape (*Brassica napus*), cabbage (*Brassica oleracea*), and horseradish (*Amoracia rusticana*), as well as the model plant *Arabidopsis thaliana*. Therefore, increased knowledge of plant pathogenic phyllosphere protists, in addition to the potential vectors and human pathogens cited above, is likely to be of economic and scientific importance. Crop species, however, are not suitable to study the natural coevolution of host plants with their microbial communities (Baldwin 2001); this is best conducted on wild close relatives. Typical wild representatives of Brassicaceae that co‐occur with *A. thaliana* (Camelineae) are spring draba (*Draba verna agg*., Arabideae), hairy bittercress (*Cardamine hirsuta*, Cardamineae), and the cockooflower (*Cardamine pratensis*, Cardamineae). All these plant species are widely distributed and commonly found in open, disturbed soil habitats, except *C. pratensis*, which mainly occurs on moist, unfertilized grasslands.

We chose a targeted cloning and sequencing approach to gain an overview on the diversity of Cercozoa on Brassicaceae. We studied *A. thaliana* and three wild relatives to investigate the potential plant specificity of cercozoans within Brassicaceae, and sampled in two distant locations to account for spatial patterns in protist community assembly. The unexpected high diversity of leaf‐associated Cercozoa, including protist plant pathogens, indicated that protists must be considered to be an integral part of the phyllosphere microbiome.

## Materials and Methods

### Sampling

Populations of four brassicaceous species (*A. thaliana* (L.) heynh., *D. verna agg*. L., *C. hirsuta* L., *C. pratensis* L.) were sampled at two distinct locations in Germany (Düsseldorf N51.188835, E6.795268; Frankfurt N50.098375, E8.546706), whereby *C. pratensis* was collected in Eschborn (N50.135553, E8.577337) instead of Frankfurt, where this species was not found. Up to three rosette leaves (three for *D. verna agg*., one to two for *A. thaliana*, one for *C. hirsuta* and *C. pratensis*) were collected from 16 individual plants per species in each location and stored in sterile 2 ml tubes for later DNA extraction. The leaves were not treated or surface‐sterilized prior DNA extraction. Care was taken not to cross‐contaminate the samples. DNA was extracted with a strongly modified protocol based on Michiels et al. (2003) (Data S1), measured using an Implen NanoPhotometer (Implen GmbH, München, Germany), and subsequently diluted to 10 ng/μl.

### Amplification and sequencing of 18S SSU gene

The amplification of 18S gene fragment was conducted with a modified version of reverse primer 1256R of Bass and Cavalier‐Smith (2004) (1256R_mod: 5′‐RDRATYAAGAAAGADCTTCAA‐3′) and a newly developed forward primer 48F_Cerco (5′‐GCCATGCAWGTCTAAGWATA‐3′). These primers were designed to specifically amplify Cercozoa and to exclude other groups of organisms, especially plants and fungi. However, due to the large diversity within this group, it was not possible to design a primer that amplified all known cercozoan genera.

Polymerase chain reaction (PCR) was conducted using Phusion High Fidelity DNA Polymerase (New England BioLabs, Ipswich, MA). The PCR reaction contained 1× Phusion GC Buffer, 200 μM dNTPs, 0.8 mg/ml BSA, 3% DMSO, 0.5 μM forward and reverse primer, 0.5 units polymerase, and 10 ng DNA (PCR conditions in Table 1). PCR reactions were conducted for the 16 individual plant‐leaf samples per species by location combination separately and pooled before cloning. The cloning reaction was conducted with StrataClone Blunt PCR Cloning Kit (Agilent Technologies, Santa Clara, CA) following the manufacturer's instructions. Clones were picked, inserts amplified with PCR primers under same conditions as described above, and PCR products were sequenced at the Labcenter of BiK‐F (Biodiversity and Climate Research Center, Frankfurt (Main), Germany).

**Table 1:** Touchdown PCR program

	*T* in °C	*t* in s	
1.	98	600	
2.	98	40	Steps 2–4 repeated for 40 times with a temperature decrement of 0.1 °C per cycle in step 3
3.	62	40	
4.	72	60	
5.	72	480	

### Sequence processing and operational taxonomic unit clustering

Forward and reverse sequences were assembled and merged using Geneious version 5.6, aligned using MAFFT version 7 (Katoh and Standley 2013; http://mafft.cbrc.jp/alignment/server/), and globally trimmed to remove terminal gaps. The detection of chimeras was carried out with mothur (Schloss et al. 2009; chimera.uchime) and the Protist Ribosomal Reference database (PR2 database, Guillou et al. 2013; http://ssu-rrna.org/, accessed November 2014) as reference. Subsequently identical sequences were removed for every species by location combination separately using usearch (Edgar 2010; derep_fulllength function). Genetic distances were calculated by mothur (pairwise.seqs) using the default options and the Needleman algorithm for alignment of the sequences. The resulting distance file was used for clustering (hcluster function of mothur) with the precision parameter set to 1,000. The output file, which contains all possible identity thresholds and its associated number of operational taxonomic units (OUTs), was used to graphically determine the optimal threshold. Subsequently, OTU consensus sequences had been calculated using mothur (consensus.seqs).

### Phylogenetic calculations

Reference sequences were obtained from PR2 database. The database file, which contained the longest sequences after 99% clustering, was chosen for analysis (available at http://ssu-rrna.org/, accessed November 2014). The five closest sequences within a 97% identity threshold, which had been determined to genus level at least, and the closest sequence including unidentified sequences within the OTU clustering threshold were extracted from the database and added to the OTU consensus sequence file for alignment and phylogenetic calculation. The sequences were aligned with MAFFT using the E‐insi algorithm. All other parameters were set to default. Phylogenetic calculations were conducted with Minimum evolution, Maximum Likelihood, and Bayesian inference algorithm using FastTree (Price et al. 2010), RAxML (Stamatakis 2014) and MrBayes (Ronquist et al. 2012) at the Trease webserver (Mishra, B., Ploch, S., Weiland, C. & Thines, M., unpubl. data).

## Results

In total, 205 sequences with a length of approximately 1 kb were obtained. Fifty‐six sequences were not affiliated to Cercozoa and excluded from further analysis, as well as three chimeric sequences. In total, 146 sequences were obtained for analyses with a minimum of 17 sequences per plant by location combination; except for *C. pratensis* from Eschborn for which initially more than 40 positive clones were sequenced, but only five contained the target fragment (Table 2). All 146 sequences used in the analysis are available in the Genebank database (http://www.ncbi.nlm.nih.gov/) under the accession numbers KT251053–KT251198.

**Table 2:** Number of different operational taxonomic units (OUTs) to which sequences were affiliated based on an identity threshold of 98.7%, number of unique sequences after dereplication, and total number of sequences larger than 1 kb

	No. of OTUs		No. of unique sequences		No. of sequences	
	Frankfurt	Düsseldorf	Frankfurt	Düsseldorf	Frankfurt	Düsseldorf
*Arabidopsis thaliana*	9	7	12	13	17	20
*Cardamine hirsuta*	9	9	14	15	20	22
*Draba verna agg*.	5	8	11	14	20	19
*Cardmine pratensis*	4^a^	8	5^a^	12	5^a^	23

a Plants were collected 6 km away from the Frankfurt location.

**Figure 1:**
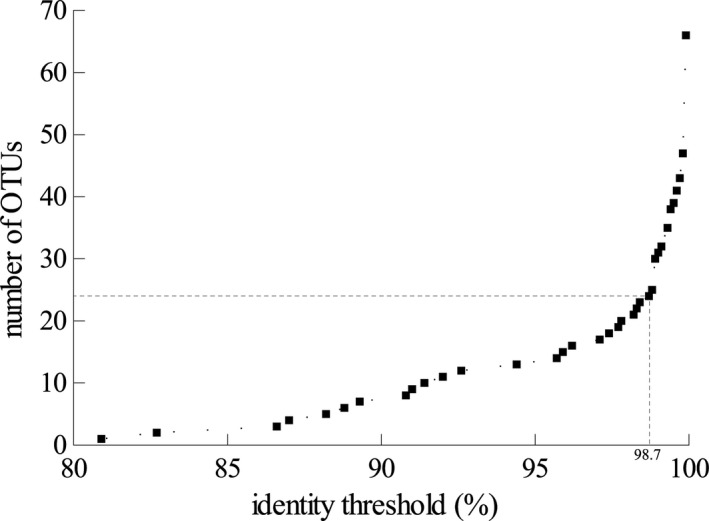
Comparison of the identity thresholds with the number of generated operational taxonomic units (OUTs). Dashed bar indicates the 98.7% identity threshold used in this analysis.

Clustering resulted in 24 different OTUs at an identity threshold of 98.7%. This threshold was determined by plotting all identity thresholds to the number of resulting OTUs (Fig. 1). For nearly all species by location combinations, up to nine distinct cercozoan taxa could be found in the phyllosphere (Table S1). Only nine taxa (37.5%) could be detected at both locations while six and nine OTUs were only detected in Frankfurt and Düsseldorf, respectively. Comparing the different plant species, only five out of 24 (20%) OTUs were shared by all four investigated species. Interestingly, *A. thaliana* had almost as many unique OTUs (29%) as all other three investigated species combined.

**Figure 2:**
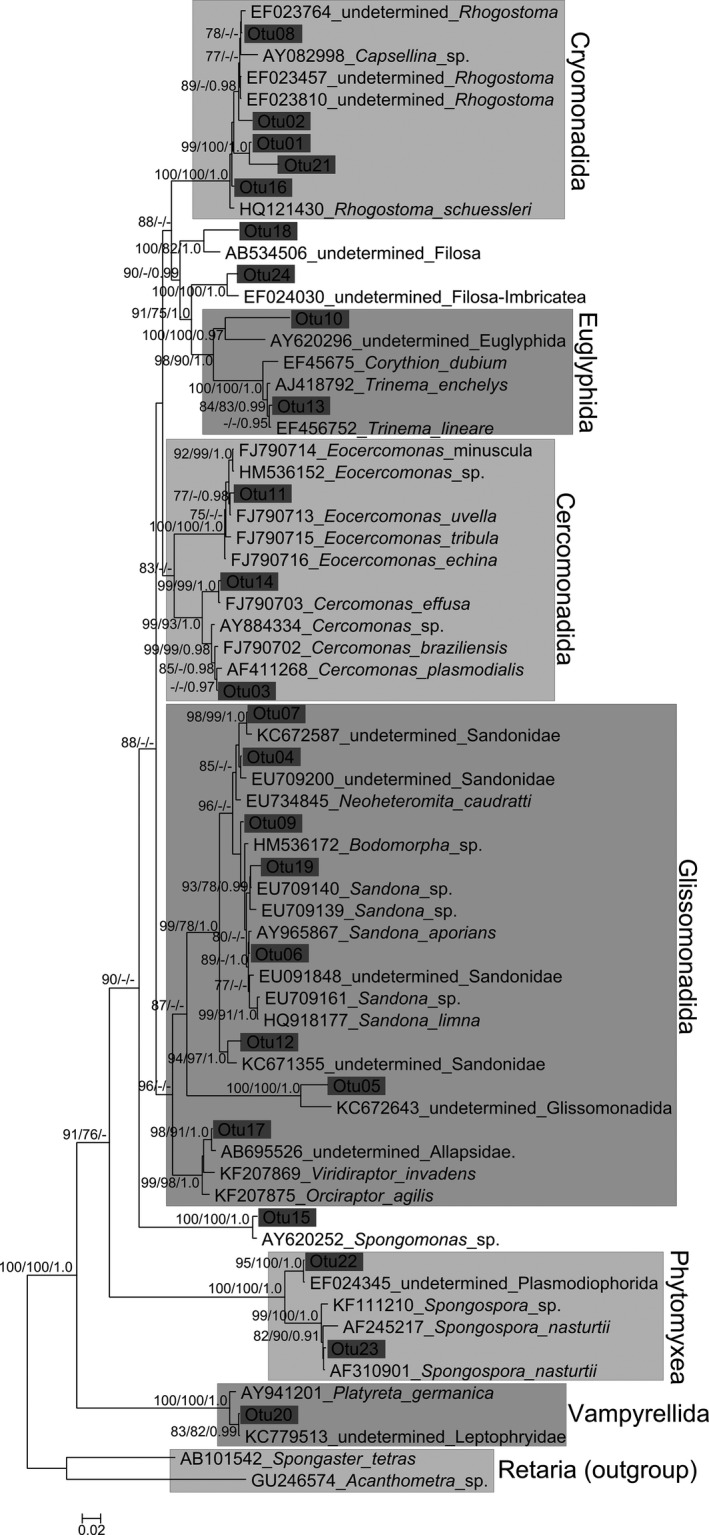
Minimum evolution tree based on 18S rDNA sequence data and calculated with Minimum Evolution algorithm. The numbers above or below the branches indicate ME‐BS, ML‐BS, or BPP values with inferior limits 75% for the bootstrap and 0.75 for the BPP analyses. Major Cercozoan groups and operational taxonomic unit (OUT) consensus sequences are indicated.

The phylogenetic tree presented in Fig. 2 uses the Minimum Evolution algorithm and includes the support values of Minimum Evolution Bootstrap (ME‐BS), Maximum Likelihood Bootstrap (ML‐BS), and Bayesian Inference posterior probability (BI‐PP). It shows a clear clustering of the major groups, although weak backbone support led to slight differences in topology between the methods (Fig. 2). These conflicts did not have any impact on the affiliation of the OTUs or the clustering of the major groups. Nearly all OTUs could be affiliated to already published sequences, but most refer to undescribed or uncultured taxa. Species of six major orders of the Cercozoa could be found to be associated to leaves within this study. Bacterivorous species from the Cercomonadida (3 OTUs), Cryomonadida (5 OTUs), and Glissomonadida (6 OTUs) represented the major fraction of detected taxa. In addition, potential phytopathogenic taxa among Phytomyxea (2 OTUs), as well as species known to feed on algae, nematodes, or fungal spores (Vampyrellidae [Endomyxa, OTU20], Viridiraptoridae [Glissomonadida, OTU17]) could be detected. Two OTUs were found in this study (OTU18, OTU24) that might represent organisms of undescribed groups.

Further phylogenetic analyses using larger sequence datasets (not shown) showed that accession KT251148 (OTU10) branched in a basal position in the euglyphid amoeba clade; accession KT251061 (OTU18) is a deep‐branching imbricate in the region of Marimonadida, Novel Clade 2, and Thaumatomonadida (Howe et al. 2011); accession KT251085 (OTU9) and KT251143 (OTU19) are sandonid glissomonads, most closely related to the *Sandona mutans* cluster (Howe et al. 2009). Accessions KT251175 and KT251078 (both OTU5) are independent detections of the same deep‐branching novel glissomonad lineage collected from the two distinct collection sites Düsseldorf and Frankfurt.

Although most OTU consensus sequences could be clustered to major groups of the Cercozoa, only 29% of the OTUs (seven out of 24) were closely affiliated to described species. All other OTUs (71%, 17 out of 24) did not form clear clusters with any sequence or were affiliated to database sequences that where either obtained from comparative environmental screenings (Bass and Cavalier‐Smith 2004; Brad et al. 2008; Howe et al. 2009; Lesaulnier et al. 2008; Nakai et al. 2012) or were byproducts of studies targeting fungi (Findley et al. 2013; Takada Hoshino and Morimoto 2010) or marine environments (Berney et al. 2013).

## Discussion

This study revealed an unexpected high diversity of cercozoan taxa on the leaves of the investigated Brassicaceae. Not even one‐third of the cercozoan sequences were assignable to described genera. The majority of sequences belonged to bacterivore, small, gliding flagellates in the Glissomonadida (Howe et al. 2009), and amoeboflagellates such as *Cercomonas and Eocercomonas* (Cercomonadida) (Bass et al. 2009b; Karpov et al. 2006). Recently, the term “phyllosphere microbiome” has been introduced to describe the diverse interacting microbial communities in and on aerial plant surfaces (Penuelas and Terradas 2014; Vorholt 2012). Considering the density and diversity of potential bacterial prey on leaves (Vorholt 2012), and the proven ability of protists to shape bacterial communities on plant surfaces (Bonkowski and Clarholm 2012; Rosenberg et al. 2009), it is surprising that protist predators up to now have been virtually absent in reviews on phyllosphere microorganisms (Andrews and Hirano 1991; Blakeman 1981; Jager et al. 2001; Kinkel 1997; Morris et al. 1996; Penuelas and Terradas 2014).

Evidence from previous studies suggests that phyllosphere protists must possess a certain suite of specific adaptations, most of all a rapid life cycle, but also the ability to form rapidly resistant cysts to survive the harsh abiotic conditions in the phyllosphere (Bamforth 1973; Mueller and Mueller 1970). As stated above, these conditions are certainly met by most of the flagellated cercozoan taxa. Since the rosettes of Brassicaceae grow close to the ground, they may be partly colonized by microbes from the underlying soil environment. For this initial study, no surface sterilization or washing of the leaves had been conducted prior DNA extraction, and it is unclear if the small, testate amoebae such as *Rhogostoma* sp. (Cryomonadida), and *Trinema* sp. (Euglyphida) in our study were true phyllosphere colonizers or must be attributed to the soil community. Especially in *A. thaliana*, the two sequences that clustered within the Viridiraptoridae and Vampyrellidae are of particular interest because their known members independently evolved mechanisms to penetrate plant cell walls (Hess and Melkonian 2013; Hess et al. 2012). A number of OTUs (OTU5, 9, 10, 18, 19, 24), which were detected for the first time here might be also potential phyllosphere colonizers. On the other hand, new lineages are found all the time, because the total diversity is still highly undersampled. However, detecting the same novel and divergent glissomonad at two different locations (accessions KT251175 and KT251078 of OTU5) in a relatively small sample size is suggestive of a protist preferentially associated with the phyllosphere. Other potential protist endophytes, including *Plasmodiophora* sp. and *Spongospora* sp. (Phytomyxea, Endomyxa), were also identified in our study.

Targeted studies with model plant species, such as *A. thaliana*,* Medicago truncatula*, and *Oryza sativa* revealed that geographic location, environmental factors, and host–plant genetic differences significantly shape the “phyllosphere microbiome” (Horton et al. 2014; Knief et al. 2010; Vorholt 2012). While host plant specificity could influence protist communities, protist communities may be more significantly affected by the biodiversity of their bacterial prey rather than the host plant per se. The low degree of overlap of taxa between the study sites Düsseldorf and Frankfurt might indicate a potential influence of geographic location as recently found for Amoebozoa (Fiore‐Donno et al. 2016), but the sequencing depth in our study was not sufficient to define any site‐ and plant‐specific patterns in bacterivore protists. Our small‐scale cloning and sequencing approach, however, did provide the first insights on the range of phyllosphere protist diversity within a single, distinct taxonomic group of protists. These data clearly confirm that protists must be considered as an integral part of the phyllosphere microbial community.

Further studies on phyllosphere protists are urgently needed. Cultivation and description of species must go hand in hand with high throughput sequencing studies to reveal the full diversity and roles of protist predators to shape the diversity, spatial structure, and function of phyllosphere bacterial communities.

## Supporting information


**Data S1.** Supplemental materials and methods.
**Table S1.** Presence/absence of OTUs for all four species at the two locations, total number of OTUs per species by location combination, and number of unique OTUs are given.
