# Supplementary material for: High Diversity Revealed in Leaf‐Associated Protists (Rhizaria: Cercozoa) of Brassicaceae
Source: J Eukaryot Microbiol. 2016 Apr 14;63(5):635–41. doi: 10.1111/jeu.12314 (PMC5031217; doi:10.1111/jeu.12314)
Supplement: Supplementary file 1 — Data S1. Supplemental materials and methods. Table S1. Presence/absence of OTUs for all four species at the two locations, total number of OTUs per species by location combination, and number of unique OTUs are given. [file JEU-63-635-s001.pdf]

SUPPORTING INFORMATION

High Diversity Revealed in Leaf Associated Protists (Rhizaria: Cercozoa) of Brassicaceae
by Sebastian Ploch, Laura Rose, David Bass, Michael Bonkowski

Supplemental Material and Methods
Supplemental Table 1 Presence/absence of OTUs for all four species at the two locations, total number of OTUs per species by location combination, and number of unique OTUs are given.

Supplemental Materials and Methods

*DNA extraction protocol based on Michiels et al. (2003) modified and adjusted for small scale extractions***.** The leaf samples were homogenized with metal beads in a 2-ml tube using a shaker mill (MM 200; Retsch GmbH, Germany). 800 µl of CTAB-buffer (100 mM Tris, 1.4 M NaCl, 20 mM EDTA, 2% CTAB, 2% Polyvinylpyrrolidone, 0.2% 2-Mercaptoethanol) was added, mixed thoroughly and incubated for 60 min at 60 °C while mixing regularly. 800 µl of phenol/chloroform/isoamyl alcohol (25:24:1; Carl Roth, Germany) was added and mixed by inverting the tube. The mixture was centrifuged for 10 min at 8,000 g, and 700 µl of the aqueous phase was transferred into a new 2-ml tube. 15 µl RNAse-A-solution (10 mg ml^{-1} RNAse A) was added and incubated at 37 °C for 15-30 min. 700 µl phenol/chloroform/isoamyl alcohol (25:24:1) was added, mixed by inverting and centrifuged as above. 600 µl of the aqueous phase was transferred to a new 2-ml tube, 600 µl chloroform/isoamyl alcohol (24:1; Carl Roth, Germany) added, mixed by inverting and centrifuged as above. 500 µl of the aqueous phase was transferred to a new 2-ml tube, 1,000 µl precipitation-buffer (100% ethanol, 150 mM sodium acetate) added, and incubated in the freezer (-20°C) for at least 1 h. After centrifugation (30 min, 4 °C, 8,000 g) the supernatant was discarded, 500 µl ice-cold washing-buffer (70% ethanol) added, and centrifuged for 5 min at 8,000 g. The washing step was repeated once, ethanol carefully removed and the pellet dried. Subsequently, the pellet was dissolved in sterile water.

Supplemental Table 1 Presence/absence of OTUs for all four species at the two locations, total number of OTUs per species by location combination, and number of unique OTUs are given.

	Düsseldorf				Frankfurt			
	A. thaliana	*C. hirsuta*	*C. pratensis*	*D. verna*	*A. thaliana*	*C. hirsuta*	*C. pratensis*	*D. verna*
OTU01	1	1	1	1	1	1	1	1
OTU02	1	1	1	0	1	1	0	1
OTU03	0	1	1	1	1	1	0	1
OTU04	1	1	1	1	1	1	0	0
OTU05	0	1	0	0	1	1	0	1
OTU06	0	1	0	1	1	0	1	0
OTU07	0	1	1	1	0	1	1	0
OTU08	0	0	0	1	0	1	0	0
OTU09	0	1	1	0	0	0	1	0
OTU10	0	0	0	0	1	0	0	0
OTU11	0	0	0	1	0	0	0	0
OTU12	0	1	0	0	0	0	0	0
OTU13	0	0	0	1	0	0	0	0
OTU14	1	0	0	0	0	0	0	0
OTU15	1	0	0	0	0	0	0	0
OTU16	0	0	1	0	0	0	0	0
OTU17	0	0	0	0	1	0	0	0
OTU18	1	0	0	0	0	0	0	0
OTU19	0	0	0	0	1	0	0	0
OTU20	1	0	0	0	0	0	0	0
OTU21	0	0	1	0	0	0	0	0
OTU22	0	0	0	0	0	1	0	0
OTU23	0	0	0	0	0	0	0	1
OTU24	0	0	0	0	0	1	0	0
total no.	7	9	8	8	9	9	4	5
unique	4	1	2	2	3	2	0	1
